# Automated parameter estimation for biological models using Bayesian statistical model checking

**DOI:** 10.1186/1471-2105-16-S17-S8

**Published:** 2015-12-07

**Authors:** Faraz Hussain, Christopher J Langmead, Qi Mi, Joyeeta Dutta-Moscato, Yoram Vodovotz, Sumit K Jha

**Affiliations:** 1 Department of Electrical Engineering and Computer Science, University of Central Florida, Orlando, Florida, USA; 2 School of Computer Science, Carnegie Mellon University, Pittsburgh, Pennsylvania, USA; 3 Department of Sports Medicine and Nutrition, University of Pittsburgh, Pittsburgh, Pennsylvania, USA; 4 Department of Biomedical Informatics, University of Pittsburgh, Pittsburgh, Pennsylvania, USA; 5 Department of Surgery and McGowan Institute of Regenerative Medicine, University of Pittsburgh, Pittsburgh, Pennsylvania, USA

**Keywords:** parameter estimation, automated parameter synthesis, computational systems biology, statistical model checking, agent-based models, acute inflammatory response

## Abstract

**Background:**

Probabilistic models have gained widespread acceptance in the systems biology community as a useful way to represent complex biological systems. Such models are developed using existing knowledge of the structure and dynamics of the system, experimental observations, and inferences drawn from statistical analysis of empirical data. A key bottleneck in building such models is that some system variables cannot be measured experimentally. These variables are incorporated into the model as numerical *parameters*. Determining values of these parameters that justify existing experiments and provide reliable predictions when model simulations are performed is a key research problem.

Domain experts usually estimate the values of these parameters by *fitting *the model to experimental data. Model fitting is usually expressed as an optimization problem that requires minimizing a cost-function which measures some notion of distance between the model and the data. This optimization problem is often solved by combining local and global search methods that tend to perform well for the specific application domain. When some prior information about parameters is available, methods such as Bayesian inference are commonly used for parameter learning. Choosing the appropriate parameter search technique requires detailed domain knowledge and insight into the underlying system.

**Results:**

Using an agent-based model of the dynamics of acute inflammation, we demonstrate a novel parameter estimation algorithm by discovering the amount and schedule of doses of bacterial lipopolysaccharide that guarantee a set of observed clinical outcomes with high probability. We synthesized values of *twenty-eight unknown parameters *such that the parameterized model instantiated with these parameter values satisfies four specifications describing the dynamic behavior of the model.

**Conclusions:**

We have developed a new algorithmic technique for discovering parameters in complex stochastic models of biological systems given behavioral specifications written in a formal mathematical logic. Our algorithm uses Bayesian model checking, sequential hypothesis testing, and stochastic optimization to automatically synthesize parameters of probabilistic biological models.

## Introduction

Over the last few years, computational modeling has emerged as a popular tool for studying and analyzing biological systems. With rapid growth in the availability of high-performance computing (HPC) infrastructure, there is increasing interest in the construction and analysis of *in silico *models of complex biological systems [[1], Chapter 5]. An essential requirement for analyzing a complex high-dimensional system is to build a sufficiently rich computational model that exhibits key properties of the real system being represented [2]. For users to have confidence in the predictions made by analyzing model simulations, it is desirable that the model be amenable to automated verification against large data-sets and expert specifications [3,4].

This process of analysis and verification becomes complicated if the system is *not deterministic*, i.e. if repeated executions of the model, under the same inputs, may produce different results. Deterministic models, while often having a clean analytic representation, cannot capture the unpredictability of natural phenomena or multi-outcome man-made artifacts. This limitation is addressed by *stochastic models *that allow a succinct representation of variability in system behavior [5]. Such models incorporate the uncertainty inherent in the system being modeled, thus facilitating more accurate analyses and predictions [6].

Models in systems biology are usually nondeterministic, nonlinear, parameterized, and describe both functional behavior and quantitative properties [7]. We will focus on a class of stochastic models that are known in the literature as *probabilistic models *[[8], Chapter 10]. The essential property of these models that is of interest to us is that it is possible to accurately assign a probability to every possible behavior that the model can exhibit [[9], §3.1].

As a case study for demonstrating our parameter estimation technique, we have considered a class of probabilistic models known as *agent-based models *(ABMs). An ABM consists of a number of autonomous, independently-acting entities known as *agents*. An agent interacts with other agents in its immediate vicinity, according to fixed rules that are possibly probabilistic, enabling the system to demonstrate behavioral variability in the face of environmental uncertainty [10,11].

An important challenge faced by designers of a biological model is to find values of unknown parameters in the model that enable it to reproduce the behavior of the relevant biological system [[12], §2.2, §3.1]. Wooley and Lin describe [[1], §5.3.3] the importance of parameter estimation in the computational modeling of biological systems:

"Identifying the appropriate ranges of parameters (e.g., rate constants that govern the pace of chemical reactions) remains one of the difficulties that every modeler faces sooner or later."

When the state-space of the parameters is small, an exhaustive search for the correct parameter values is feasible. For high-dimensional models, brute-force methods are unlikely to terminate in sub-exponential time and hence are prohibitively expensive [13].

We address this problem by designing an algorithmic technique for parameter estimation in stochastic biological models that ensures that the synthesized model conforms to desired behavior as expressed in a formal temporal logic [14]. This paper makes the following contributions:

• We describe a *new algorithm for automatically discovering parameters of probabilistic computational models *of biological systems. Our algorithm uses simulated annealing [15] and Bayesian statistical model checking [16] to efficiently explore the system's parameter space while continually verifying whether the model instantiated with the current parameters satisfies the given expert specifications.

• We demonstrate the effectiveness of our approach by applying our algorithm to automatically synthesize *twenty eight *parameters in an agent-based, physiological model of the acute inflammatory response to endotoxin administration [17].

## Related work

This section surveys major recent research results on parameter estimation in systems biology. We first summarize techniques that rely primarily on reformulating estimation as a non-linear optimization problem. Later, we discuss approaches based on formal verification.

### Parameter estimation using global and local search

Sun et al. [18] survey metaheuristic techniques used in parameter estimation in systems biology, focusing on simulated annealing, evolutionary algorithms and hybrid strategies that combine multiple heuristics.

Gonzalez et al. [19] use simulated annealing [20] to find parameters in S-system models of biochemical networks. At a given parameter point during the annealing process, they find a neighboring point by adding a noise term to *each component *of the parameter vector that is dependent on the current optimization error.

Lillacci and Khammash have designed a method that uses Kalman filtering, statistical testing and numerical optimization for parameter estimation and model selection [21]. Inspired by these two approaches [19,21], we have used simulated annealing for optimization and statistical hypothesis testing-based verification in our parameter estimation algorithm for probabilistic models.

Algorithms based on maximum-likelihood estimation (MLE) and the singular value decomposition (SVD) have been proposed by Reinker et al. [22], to estimate the reaction rate constants (the parameters) from discrete time series data for *molecule counts *in stochastic biochemical reactions.

Rodriquez-Fernandez et al. use a hybrid approach for parameter estimation in deterministic, non-linear models of biochemical pathways [23]. Their approach combining local and global optimization methods helps overcome the problem of convergence to local minima common in traditional local optimization methods (like gradient-descent) and the problem of slow convergence seen in global optimization techniques.

Moles et al. have studied the global optimization based approaches to the parameter estimation problem in nonlinear dynamic systems using a 36 parameter dynamic pathway model as a benchmark [24]. They report that only approaches based on evolutionary strategies, viz. the stochastic ranking evolutionary strategy (SRES) and unconstrained evolutionary strategy (use), were able to successfully find parameters in the pathway.

The parameter estimation problem for biological pathway models has also been addressed by Koh et al. [25]. An interesting element of their approach is to decompose the model into components whose parameters can be estimated independently. They model pathways using hybrid Petri nets and use evolutionary strategies for parameter estimation, but their approach can be used for any modeling framework and choice of the optimization technique.

Matthew et al. have used global sensitivity analysis (GSA) to study the effect of parameter perturbations in a large, non-linear, ODE-based model of acute inflammation in mice [26], and found that Interleukin 6 (IL-6) and nitric oxide (NO) had a significant, non-linear impact on inflammatory damage [27].

In another approach that uses sensitivity analysis, Donze et al. have developed an algorithm for parameter synthesis in nonlinear systems of ODEs (ordinary differential equations) [28], and applied their technique to find parameters in two models of acute inflammation [29,30].

Next, we discuss parameter estimation approaches based on formal verification techniques.

### Parameter estimation using formal verification

Many recent procedures for parameter estimation make use of a formal verification technique known as *model checking*.

A model checking approach to finding parameters in biological models has been used by Calzone et al [31]. They use the Biochemical Abstract Machine (BIOCHAM) modeling framework to describe the system and temporal logic model checking [32] to find parameters values in a user-specified range.

Dreossi and Dang have recently designed an algorithm that reduces the problem of parameter synthesis in polynomial (discrete-time) dynamical systems [33] to solving a set of linear programs [34]. They use their technique to find parameters of two well-studied epidemic models.

Batt et al. have used symbolic model checking to find parameters in a piecewise affine differential equation (PADE) model of the gene regulation IRMA network [35]. IRMA stands for *in vivo "benchmarking" of reverse-engineering and modeling approaches *[36]. Donaldson and Gilbert have designed a technique for parameter estimation that combines model checking with a genetic algorithm [37], and used it for estimating kinetic rate constants in a model of the mitogenactivated protein kinases (MAPK) signaling pathway.

Usually, model checking based methods of parameter estimation require that the relevant specification be expressed in a formal temporal logic, whose satisfaction against a given execution path (known as a *trace*) of the model can be determined. Rizk et al. define a *continuous degree of satisfaction *of a temporal logic formula for any given trace of the model [38] and use it as a fitness function to drive an optimization-style search for kinetic parameters in models of the cell cycle and the MAPK signal transduction.

In recent work, Mancini et al. have used statistical model checking to synthesize parameters in an ODE-based biological model [39]. They have implemented a distributed, multi-core version of their algorithmic technique, and used it to estimate *patient-specific *parameters of a a human menstrual cycle model [40].

## Background

Before describing our algorithm for synthesizing parameters in probabilistic models, we give some back-ground on stochastic modeling in systems biology, specification of time-varying properties using temporal logic and statistical verification of probabilistic systems against behavioral specifications. We also provide definitions for formal concepts that will be used to describe our algorithm.

*Remark *1 One of our main objectives in this section is to develop a formal definition of computational probabilistic models: i.e. those stochastic models for which *a probability can be assigned to any observed model behavior *when the model is executed. The reader familiar with this concept can quickly skim through the next two subsections.

### Stochastic biological models

Many biological systems have traditionally been described using *deterministic, mathematical *models, often in terms of ordinary differential equations [[12] §2.1]. However, in recent years, the need for incorporating the uncertainty inherent in biological systems has led to the development of *stochastic models*: these are harder to analyze but more accurately reflect the behavior of the underlying system [5]. Common stochastic models in biology include Markov jump processes [5], stochastic differential equations [41], discrete-time Markov chains [[42], Chapter 3] and continuous-time Markov chains [43].

Also, researchers are increasingly developing *computational *models that naturally capture the bottom-up nature of biological phenomena and hence are more amenable to *in-silico *implementation [44]. Such models are constructed by observing large sets of timeseries data, combined with their expert insight into the system being modeled [45]. Some of these models are based on experimental data of varying veracity and error propagation into the designed model is an ever-present challenge [46].

We will focus on *discrete-time Markov chains*, a class of stochastic models that are widely used in the sciences, engineering, economics and other areas. We closely follow Baier and Katoen [8] in formally defining them.

**Definition 1 **(Discrete-time Markov chain) A discrete time Markov chain (DTMC) is given by M=(S,P,init,AP,L) where:

• *S *is a countable, nonempty, set of states,

• *init *: *S *→ [0, 1] is the initial distribution such that $\sum_{s\in S}init\left(s\right)=1$,

• *P *: *S *× *S *→ [0, 1] is the transition probability function,

• *AP *is a set of boolean valued atomic propositions,

• *L *: *S *→ 2*^AP ^*is a labeling function for states. ■

We next define (a) a parameterized family of DTMCs, given a set of parameters and (b) execution paths over them.

**Definition 2 **(Parameterized DTMC) A parameterized discrete time Markov chain (ParDTMC) is given by $M=\left(S,\Theta_{m},P,init,AP,L\right)$ where

• *S *is a countable, nonempty, set of states,

• $\Theta_{m}={\mathbb{R}}^{m},\;m\geq 1$ is the parameter space,

• *init *: *S *→[0, 1] is the initial distribution such that $\sum_{s\in S}init\left(s\right)=1$,

• *P *: *S *× *S *× Θ*_m _*→[0, 1] is the transition probability function over an *m*-dimensional parameter space,

• *AP *is a set of boolean valued atomic propositions, and

• *L *: *S *→ 2*^AP ^*is a labeling function for states. ■

**Definition 3 **(ParDTMC execution paths) An execution path of a ParDTMC $M=\left(S,\Theta_{m},P,init,AP,L\right)$ given by *σ *= *s*_0_, *s*_1_, . . .. The suffix of a path *σ *that starts at state *s_i _*(i.e. *s_i_, s*_*i*+1_, . . .) is denoted *σ^i^*.

As a case study, we have applied our automated parameter estimation technique to an agent-based model of the acute inflammatory response. We now briefly discuss major characteristics of ABMs.

### Agent-based modeling in systems biology

Agent-based models (ABMs) form an interesting, wellstudied subset of complex probabilistic models. In recent years, agent-based modeling has emerged as a popular method for the representation, analysis and simulation of biological models [10].

ABMs allow the specification of high-level model properties as well as fine-grained component behavior. An ABM is composed of autonomous elements whose individual properties can be specified. At a macro-level, model-wide agent-interaction rules can be defined and enforced. Each agent has a physical location and its state evolves with time based on messages exchanged with other agents, allowing rich spatiotemporal properties to emerge [10].

ABMs can incorporate parallelism, object-oriented behavior and stochasticity, making them conducive to the development of computational models in systems biology. Another advantage of using ABMs is that they are *bottom-up *models that mirror the natural behavior of biological systems [47]. Agents interact with one another based on fixed rules and also have a spatial location [10].

Most agent-based models do not have compact analytic descriptions, and simulations across the input space must be performed to in order to infer general model properties [48]. Also, agent-based models tend to have a large number of parameters, some of them highly-sensitive in the sense that a small change can radically alter model behavior [49]. To the best of our knowledge, the parameter estimation problem in ABMs has only been addressed for very simple models [50] and most approaches use standard optimization techniques [31,51].

As a case study for our parameter estimation technique, we have used an agent-based model of the acute inflammatory response due to endotoxin administration [52] written using the SPARK tool [53]. SPARK is an ABM framework designed for multi-scale modeling of biomedical models that is implemented in Java and allows users to develop models using its own programming language (SPARK-PL) [54-57].

One we have a basic model design available, we need to find the *exact model instantiation *in terms of concrete parameter values that meets desired behavior (usually experimental data). Therefore, we need a way to check if the models all requirements. We now discuss how to *formally specify *and *automatically verify *probabilistic computational models.

An ABM  A consists of a fixed number of agents *A*_1_, . . . *A_n_*, where the state of agent *A_i _*is determined by the values of variables $V_{1}^{i}\cdots V_{m}^{i}$. Assuming that for any agent *A_i_, i *∈ {1 . . . *n*}, variable $V_{j}^{i}$, *j *∈ {1 . . . *m*} can take values in the set *V ar_j_*,  A can be represented as a DTMC.

**Definition 4 **(DTMC corresponding to an ABM) Consider ABM A(n,m) with *n *agents A_1_, . . . *A_n _*and *m *variables *V*_1 _. . . *V_m _*that take values over countably finite sets *V ar*_1_, . . ., *V ar_m _*respectively. The DTMC $M_{A}=\left(S,P,init,AP,L\right)$ corresponding to A(n,m) is:

• $S={\left(V\;ar_{1}\times \ldots \times V\;ar_{m}\right)}^{n}$

• *init *: *S *→[0, 1], the initial distribution is imposed by  A,

• *P *: *S *× *S *→[0, 1] is determined by the transition rules over variables of  A,

• *AP *consists of (boolean-valued) atomic propositions over agent variables $V_{1}^{1}\ldots V_{m}^{1},\;\ldots ,\;V_{1}^{n}\ldots V_{m}^{n}$ and

• *L *: *S *→ 2*^AP ^*is the usual function marking each state with propositions that hold there. ■

We generalize agent-variable ABMs, by adding the notion of parameters to obtain a *family *of ABMs. More formally, we talk of a parametric ABM A(n,m,k) over *n *agents, *m *variables and *k *parameters whose dynamic behavior (i.e. its transition function *P*) now also depends on the parameter values. For a given parametric ABM, we can defined an equivalent ParDTMC.

**Definition 5 **(ParDTMC corresponding to a parametric ABM) Consider ABM A(n,m,k) with *n *agents *A*_1_, . . . *A_n _*and *m *variables *V*_1 _. . . *V_m _*that take values over countably finite sets *V ar*_1 _. . . *V ar_m _*and *k *parameters *θ*_1 _. . . *θ_k_*. The ParDTMC $M_{A}=\left(S,\Theta_{k},P,init,AP,L\right)$ corresponding to A(n,m,k) is:

• $S={\left(V\;ar_{1}\times \ldots \times V\;ar_{m}\right)}^{n}$,

• *init *: *S *→[0, 1] the initial distribution is imposed by  A,

• *P *: *S *× *S *× Θ*_k _*→[0, 1], where Θ*_k _*= *θ*_1 _× . . . × *θ_k _*is determined by the transition rules over variables and parameters of  A,

• *AP *consists of boolean propositions over variable values of all agents $V_{1}^{1}\ldots V_{m}^{1},\;\ldots ,\;V_{1}^{n}\ldots V_{m}^{n}$,

• *L *: *S *→ 2*^AP ^*is the usual function marking each state with propositions that hold there. ■

*Remark *2 In order to use statistical model checking to solve the probabilistic model checking problem, we need to associate probabilities with executions paths of the model. For DTMCs, this has been shown by Kwiatkowska et al [9] and Baier and Katoen [[8], Chapter 10]. Although we do not prove this, this is also true for ParDTMCs. From now on, we assume that our models can be represented as ParDTMCs and thus have a unique underlying probability measure.

### Specifying and checking biological properties

Our algorithm discovers parameters of stochastic biological from experimental data and expert behavioral specifications. While exploring the parameter space, we continually verify whether we have a parameter assignment at which the model meets the given specifications.

Typically, properties of biological systems that need to be formally specified are *qualitative, quantitative *and *time-varying*. Since the models are usually stochastic, specifications too should allow reasonable deviations from desired behavior. To capture such rich behavioral properties, we use a *probabilistic temporal logic *(see Definition 8) to specify expected model behavior.

In order to develop automated techniques for analyzing biological models, we also need the properties to be specified in a way that can be *monitored *as we perform ABM simulations. Monitoring is the process of determining if an execution trace of the model satisfies given specifications [58,59]. Therefore, we write specifications using a logic belonging to a class of languages for which monitors can be derived algorithmically [60].

We translate natural language expert insights representing desired model behavior into a logic whose sentences are *adapted finitely monitorable *(AFM) formulas. The truth value of an AFM formula with respect to a trace of a model execution can be determined by observing a finite prefix of the trace [[61], §2],[[60], §2.2]. Next, we formally define our specification language:

**Definition 6 **(BLTL grammar) Given a ParDTMC $M=\left(S,\Theta_{k},P,init,AP,L\right)$, the grammar of bounded linear temporal logic (BLTL) formulas *φ *in Backus-Naur Form is as follows:

$\left\langle \phi \right\rangle ::=a|\phi \wedge \phi |\phi \vee \phi |\neg \phi |\phi {\text{U}}^{d}\phi$

where *a *∈ *AP*, d∈ℕ, and ∧, ∨ and ¬ are the usual proposition logic operators. We call **U**^*d*^ the bounded until operator. ■

The semantics of a BLTL formula *φ *is defined over an execution path of a ParDTMC  M (see Definition 3).

**Definition 7 **(BLTL semantics) Given a path *σ *of a ParDTMC M=(S,P,init,AP,L), the satisfaction of a BLTL formula *φ *on a path suffix $\sigma^{i}=\left(s_{1},\;s_{2},\;\ldots \right)$ is denoted $\sigma^{i}⊨\phi$, and is determined by the following rules:

• *σ^i ^*⊨ *a*, where *a *∈ *AP *iff *a *∈ *L*(*s_i_*),

• *σ^i ^*⊨ *φ*_1 _^ *φ*_2 _iff *σ^i ^*⊨ *φ*_1 _and *σ^i ^*⊨ *φ*_2_,

• *σ^i ^*⊨ *φ*_1 _∨ *φ*_2 _iff *σ^i ^*⊨ *φ*_1 _or *σ^i ^*⊨ *φ*_2_,

• *σ^i ^*⊨ ¬*φ *iff *σ^i ^*⊭ *φ*,

• $\begin{array}{c} \sigma^{i}⊨\phi_{1}U^{d}\phi_{2}\text{iff }\exists l\in \mathbb{N}\;\text{such}\;\text{that}\;l<=d,\;\sigma^{i+1}⊨\phi_{2}\;\\ \text{and}\;\forall 0\leq j<l,\;\sigma^{i+j}⊨\phi_{1}.■ \end{array}$

We now state, without proof, the fact that it is possible to check if any path of a ParDTMC satisfies a given BLTL formula, by observing a finite prefix of the path. For a proof of a similar lemma for continuous-time Markov chains, see Legay et al [62].

**Lemma 1 **(Monitorability of BLTL formulas over DTMCs) *It is always possible to algorithmically decide if any simulation path σ *= (*s*_1_*, s*_2_, . . .) *of a ParDTMC * M*satisfies given BLTL formula φ by observing a finite prefix of σ*.

*Note *1 (Additional bounded operators) We can define bounded versions of the usual linear temporal logic operators **G **(*always*) and **F **(*eventually*) as follows: **F**^*d*^*ψ = ***true U**^*d*^*ψ*, **G**^*d*^*ψ *= ¬**F***^d^*¬*ψ*. **F***^d^ψ *means that *ψ *holds at some state within the next *d *state-transitions, and **G**^*d*^*ψ *means that *ψ *continually holds for the next *d *state-transitions.

The expressive power of our agent-based models emanates from their ability to capture uncertainty. For such stochastic models, a single execution trace that violates a given property cannot serve as a counterexample. We need a more flexible specification language whose formulas allow reasonable deviations from expected model behavior. For this, we define a probabilistic version of BLTL.

**Definition 8 **(Probabilistic Bounded Linear Temporal Logic) A specification of the form *P_≥ θ _*(*φ*) is a probabilistic bounded linear temporal logic (PBLTL) formula if *φ *is a bounded linear temporal logic formula and *θ *is a probability threshold such that θ∈ℝ, 0 ≤ *θ *≤ 1. If a *θ *fraction of the traces of a model satisfy *φ*, we deem the model to satisfy the (probabilistic) AFM specification *P_≥ θ _*(*φ*).■

### Automated verification using model checking

Model checking is an automated technique for verifying finite and infinite state transition systems that is widely used for formal assurance of safety-critical systems [8]. Techniques based on model checking have been used for verification in a number of areas such as hybrid dynamical systems [63], computer software [64] and systems biology [43].

In order to use model checking to verify a time-varying system, the model is described using a Kripke structure and the property to be checked is written in a formal temporal logic [32]. We will use PBLTL (Definition 8) to define the probabilistic model checking problem.

**Definition 9 **(Probabilistic model checking (PMC)) Given a probabilistic model  M and a PBLTL specification *P_≥ θ _*(*φ*), determine if  M satisfies *φ *with probability at least *θ*. ■

There are two approaches for solving the PMC problem:

• Symbolic and numerical techniques [65,66] that estimate the exact value of the probability with which  M satisfies *φ *(by exhaustively exploring all possible model behaviors) and then compare it to the specification threshold probability *θ*.

• Statistical techniques [67-70] that use a set of sample simulations to determine if $M⊨P_{\geq \theta }\left(\phi \right)$.

Statistical approaches for probabilistic model checking are more scalable since they avoid the expensive calculation associated with accurately estimating exact probabilities. However, a limitation of statistical model checking algorithms is that the reported result is not guaranteed to be correct, i.e., there may be false positives or false negatives [71]. For an excellent overview of major statistical model checking techniques, we refer the reader to Legay et al [61].

Since computational models in systems biology often have large parameter spaces, we use statistical model checking as part of our parameter estimation algorithm. Next, we describe a reformulation of the probabilistic model checking problem in terms of statistical hypothesis testing - an approach first used by Younes [71].

### Hypothesis testing based statistical model checking

We are interested in determining an answer to the probabilistic model checking problem, i.e. "Does $M⊨P_{\geq \theta }\left(\phi \right)$?" (see Definition 9).

Assuming that the model's actual probability of satisfying the specification is *u *∈[0, 1], i.e. $M⊨P_{=u}\left(\phi \right)$, we test the (null) hypothesis *H *: *u ≥ θ *against the (alternative) hypothesis *K *: *u *<*θ*. If *K *is rejected we conclude that $M⊨P_{\geq \theta }\left(\phi \right)$. If *H *is rejected we conclude that $M⊭P_{\geq \theta }\left(\phi \right)$.

*Note *2 (Errors in hypothesis testing) For a hypothesis test procedure, the critical region is the part of the sample space where the null hypothesis is rejected. If the test rejects *H *when its true, it is considered a type-I error. On the other hand, if the test accepts *H *when its false, it is a type-2 error. Once the critical region for a test procedure has been decided, it uniquely determines the probabilities of type-1 and type-2 errors. For a given critical region, we denote by *α *(resp. *β*) the probability of a type-1 (resp. type-2) error [[72], §1.3].■

Naturally, for any statistical hypothesis testing procedure we want a critical region that minimizes the probabilities *α *and *β *of Type-1 and Type-2 errors (see Note 2). However, this implies either using a large value for either *α *or *β*, or drawing a large number of samples to ensure test accuracy.

Younes [70] suggests that the solution is to use the more relaxed test of *H*_0 _: *u *≥ *u_r _*against *H*_1 _: *u *≤ *u_l_*, where 0 ≤ *u_l _*<*θ *<*u_r _*≤ 1. If *H*_0 _(*H*_1_) is accepted, we consider *H *(resp. *K*) to be true.

*Remark *3 (Indifierence regions) [*u_l_, u_r_*] is known as the indifierence region. If *u *∈ [*u_l_, u_r_*] (i.e. when both *H*_0 _and *H*_1 _are false), we do not care about the result of the test; thus the test procedure is allowed to accept either hypothesis. In practice, we often choose the indifierence region [*u_l_, u_r_*] to be of width 2 * ∈ (where 0<∈≪1), i.e. [*u *- ∈, *u *+ ∈].

Our parameter estimation technique uses the Bayesian statistical model checking (BSMC) algorithm developed by Jha at al [16], to algorithmically check if a probabilistic model satisfies given behavioral specifications. Note that a fully Bayesian statistical model checking algorithm that verifies Dynamic Bayesian Networks against probabilistic queries was developed by Langmead [73].

Below, we briefly describe the main idea behind BSMC and refer to the reader to the literature for details [60,61,16].

### Bayesian statistical model checking

Recall that we are interested in testing whether a probabilistic model meets a PBLTL specification with a minimum threshold probability, i.e. "Does $M⊨P_{\geq \theta }\left(\phi \right)$?"

Assuming that $M⊨P_{=u}\left(\phi \right)$, we have posed this problem as hypothesis testing query: test *H *: *u *≥ *θ *against *K *: *u *<*θ*, and then relaxed it to use indifference regions: *H*_0 _: *u *≥ *u_r _*against *H*_1 _: *u *≤ *u_l _*(where 0 ≤ *u_l _< θ < u_r _*≤ 1).

If *H*_1 _is rejected, we consider that H : *u *≥ *θ *holds, and hence conclude that $M⊨P_{\geq \theta }\left(\phi \right)$. If *H*_0 _is rejected, we conclude that $M⊭P_{\geq \theta }\left(\phi \right)$, i.e. $M⊨P_{<\theta }\left(\phi \right)$.

Recall that we do not know the value of the actual probability *u *with which the model satisfies the specification i.e. $M⊨P_{=u}\left(\phi \right)$. For Bayesian testing, we model this unknown probability as a random variable *U *and assume the availability of a *prior density *function *g *(.) that incorporates our existing knowledge of *U*.

The Bayesian statistical model checking procedure (see Algorithm 1) sequentially draws traces from  M in an i.i.d. fashion until it rejects either *H*_0 _or *H*_1_. For each sample trace *σ_i_*, (*i *∈ {0, 1, . . .}) of  M, it uses a monitoring algorithm to determine if the path satisfies the BLTL specification *φ*.

The outcome of each such test of path satisfiability can be represented by a Bernoulli random variable *X_i _*i.e. $\forall_{i}\in \left\{1,\;...n\right\}$, if $\sigma_{i}⊨\phi$, *X_i _*= 1, otherwise (i.e. when $\sigma_{i}⊭\phi$) *X_i _= *0. At each iteration, the algorithm calculates a quantity known as the Bayes Factor (*BF*) that, given the observation of a sample {*x*_1_, *x*_2_, . . . *x_n_*}; *x_i _*∈ {0, 1}, reflects confidence in *H*_0 _holding versus *H*_1_:

(1) $$BayesFactor:\frac{P\left(x_{1},\;\ldots ,\;x_{n}|H_{0}\right)}{P\left(x_{1},\;\ldots ,\;x_{n}|H_{1}\right)}$$

**Algorithm 1 **Bayesian statistical model checking (BSMC) algorithm for probabilistic models

Require:

Probabilistic model  M,

PBLTL specification $P_{\geq \theta }\left(\phi \right)$,

Threshold *L *> 1,

Prior density function *g*(·) of the unknown parameter *u *where $M⊨P_{=u}\left(\phi \right)$

Indifference region bounds: (*∈*_1_*, ∈*_2_), where *∈*_1 _> 0, *∈*_2 _> 0.

{Note: Indifference region is [*θ *- *∈*_1_, *θ *+ *∈*_2_]

{Note: *H*_0 _: *u ≥ θ *+ *∈*_2_; *H*_1 _: *u ≤ θ *- *∈*_1_}

Ensure:

*ans *= **false **if *H*_0 _rejected,

*ans *= **true **if *H*_0 _is accepted,

*n *= Total number of traces sampled from  M.

1: *n *← 0 {Number of traces drawn from  M.}

2: *z *← 0 {Number of traces satisfying *φ*.}

3: **repeat**

4: Draw sample *σ *from  M

5: *n *← *n *+ 1

6: **if ***σ ***⊨ ***φ ***then**

7: *z *← *z *+ 1

8: **end if**

9: $B\leftarrow \frac{\int_{\theta +\in_{2}}^{1}u^{z}{\left(1-u\right)}^{n-z}g\left(u\right)du}{\int_{0}^{\theta -\in_{1}}u^{z}{\left(1-u\right)}^{n-z}g\left(u\right)du}$ (2)

10: **until $\left(B>L\right)\vee \left(B<\frac{1}{L}\right)$**

11: **if ***B *>*L ***then**

12: *ans *← *true*

13: **else**

14: *ans *← *f alse*

15: **end if**

16: **return **(*ans*; *n*)

We need to calculate the probability *P*(*d*|*H_i_*) of observing sample *d *= (*x*_1_, *x*_2_, . . . *x_n_*) given hypothesis *H_i_, i *∈ {0, 1}. Therefore, we will need to consider all cases where *H*_0 _(resp. *H*_1_) holds. Assuming the indifference region [*u_l_, u_r_*] to be [*θ *- ∈_1_, *θ *+ ∈_2_], whether *H*_0 _: *u ≥ u_r _*or *H*_1 _: *u ≤ u_l _*holds depends on the actual probability *u *of  M satisfying *φ*. For both cases, we integrate over all possible values of *u *according to our prior *g*:

$$P\left(d|H_{0}\right)=\int_{\theta +\in_{2}}^{1}f\left(x_{1}|u\right)\ldots f\left(x_{n}|u\right)g\left(u\right)du$$

$$P\left(d|H_{1}\right)=\int_{0}^{\theta -\in_{1}}f\left(x_{1}|u\right)\ldots f\left(x_{n}|u\right)g\left(u\right)du$$

Here, $f\left(x_{i}|u\right)=u^{x_{i}}{\left(1-u\right)}^{1-x_{i}}$ is the conditional density of Bernoulli random variable *X_i _*given the actual probability *u*. We thus obtain the following expression for the Bayes factor:

$$BayesFactor=\frac{\int_{\theta +\in_{2}}^{1}u^{z}{\left(1-u\right)}^{n-z}g\left(u\right)du}{\int_{0}^{\theta -\in_{1}}u^{z}{\left(1-u\right)}^{n-z}g\left(u\right)du}$$

In summary, calculating the Bayes factor requires knowing the number of total traces n drawn from  M, the number *z *of traces that satisfied specification *φ*, the indifference region [*u_l_, u_r_*] and the prior density *g*(·) of the unknown probability *u*. For more details about the Bayesian model checking algorithm, we refer the reader to the work of Jha [[60], Chapter 4].

## Algorithm for discovering parameters

We formally state the problem of estimating parameters in computational models of probabilistic systems:

**Definition 10 **(Parameter estimation problem) Given a parameterized probabilistic model M(ω), with parameter set $\Omega \subseteq {\mathbb{R}}^{n}$, a desired specification *φ *in a bounded (finitely monitorable) temporal logic, and a probabilistic threshold *θ *∈[0, 1], find a parameter value *ω *∈ Ω such that M(ω) satisfies property *φ *with probability at least *θ*, i.e. $M\left(\omega \right)⊨P_{\geq \theta }\left(\phi \right)$. ■

Our algorithmic procedure for synthesizing parameters is shown in Algorithm 2. We use simulated annealing [74] for exploring the parameter space of the stochastic model. Simulated annealing is a stochastic optimization technique that avoids local minima in two ways (lines 17-27): (a) by sometimes accepting points with lower fitness and (b) via the temperature schedule that causes fewer bad choices to be accepted as we move closer to one of the global optima.

When considering any candidate parameter *ω *∈ Ω, we invoke the Bayesian model checking routine (lines 2 and 12) to check if the *parameterized model *matches expected behavior (i.e. if $M\left(\omega \right)⊨P_{\geq \theta }\left(\phi \right)$).

Note that since the BSMC algorithm expects as input a prior density *g *for the unknown probability *u *where $M⊨P_{=u}\left(\phi \right)$, our synthesis algorithm needs a parameterized prior *h*(·) that represents the prior for each model instantiation M(ω).

**Figure 1:**
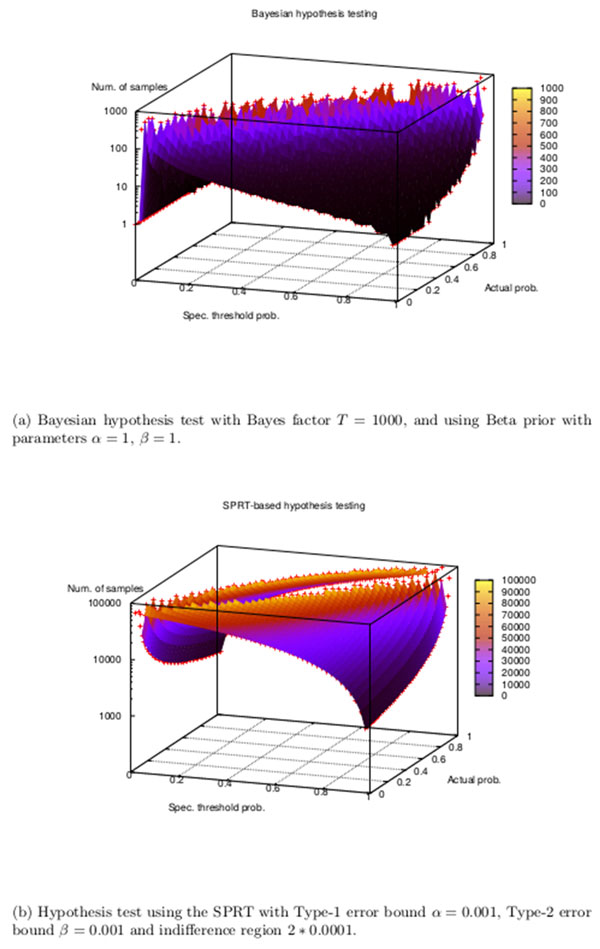
**Comparison of the efficiency of (a) Bayesian and (b) SPRT hypothesis testing**. In both cases, the number of samples required for hypothesis testing increases as the specification threshold probability approaches the actual probability with which the model satisfies the specification. Bayesian hypothesis testing required fewer samples than the SPRT when the model is obviously flawed with respect to the desired behavior. The number of samples for the Bayesian hypothesis testing vary from 1 to 1000 while those for the SPRT become as large as 100000.

To guide the annealing process, we use the number of samples returned by the Bayesian model checking procedure, moving to the parameter point that needed *more samples to reject the null hypothesis during Bayesian statistical model checking *(lines 17 to 20 in Algorithm 2). For verifying a model  M against a PBLTL formula $P_{\geq \theta }\left(\phi \right)$, given that  M actually satisfies *φ *with probability *u *(i.e. $M⊨P_{=u}\left(\phi \right)$), the Bayesian statistical model checking algorithm takes increasingly larger number of samples to reject the null hypothesis as the specification threshold probability (*θ*) approaches the actual probability (*u*) with which  M satisfies *φ*, as shown in Figure 1. The figure shows how, for a fixed threshold *θ*, the Bayesian hypothesis testing algorithm takes a larger number of samples for verification when we consider parameter points *ω *at which the model's probability *p*(*ω*) of satisfying *φ *is close to *θ*.

In earlier work, we had demonstrated the use of statistical hypothesis testing for parameter search in stochastic models by using a metric based on the Sequential Probability Ratio Test (SPRT) hypothesis testing technique [75] as a fitness function to drive the global optimization procedure used for searching the state space [76,77].

**Algorithm 2 **Parameter estimation for probabilistic models using Bayesian statistical model checking

Require:

Parameterized probabilistic model M(⋅) on parameter space Ω,

PBLTL specification $P_{\geq \theta }\left(\phi \right)$,

Starting temperature *t_s_*,

Stopping temperature *t_f_*,

Cooling schedule *T *: ℕ↦[0,∞) (where *T *is strictly decreasing),

Parameterized prior density *h*(·) on paramerter space Ω, Threshold *L*,

Indifference region bounds (*∈*_1_, *∈*_2_) where *∈*_1 _> 0, *∈*_2 _> 0.

Ensure:

ans = *ω *such that *ω *∈ Ω and $M\left(\omega \right)⊨P_{\geq \theta }\left(\phi \right)$ or

ans = "No parameter found."

1: *ω *← an element in Ω selected randomly

2: (*f, n*) ← *BSMC *$\left(M\left(\omega \right),P_{\geq \theta }\left(\phi \right),\;L,\;h\left(\omega \right),\;\left(\in_{1},\;\in_{2}\right)\right)$

3: **if ***f *= *true ***then**

4:    *ans *← *ω*

5:    **return**

6: **end if**

7: *t *= *t_s_*

8: *lcount *= 0

9: **while ***t ≥ t_f _***do**

10:    *lcount *← *lcount *+ 1

11:    Select a neighbor ω′ of *ω *randomly.

12:    $\left(f\prime ,\;n\prime \right)\leftarrow BSMC\left(M\left(\omega^{\prime }\right),P_{\geq \theta }\left(\phi \right),\;L,\;h\left(\omega \right),\;\left(\in_{1},\;\in_{2}\right)\right]$

13:    **if **f′=true**then**

14:        ans←ω′

15:        **return**

16:    **end if**

17:    if n′>n**then**

18:        ω←ω′

19:        f←f′

20:        n←n′

21:    **else**

22:        **if ***rand*(0, 1) >exp(-(n′-n)/t)**then**

23:            ω←ω′

24:            f←f′

25:            n←n′

26:        **end if**

27:    **end if**

28:    *t *← *T *(*lcount*)

29: **end while**

30: *ans *← "No parameter found"

31: **return ***ans*

In Figure 1, we show results from two sets of experiments that demonstrate how Bayesian hypothesis testing uses significantly fewer samples than a verification procedure based on the SPRT when the model is far away from the desired behavior. Unlike our earlier technique for parameter discovery [77], Algorithm 2 does not need *the Type-1, Type-2 error bounds *(*α, β *resp.) because it uses Bayesian statistical model checking for verification, thereby reducing the number of samples required for discovering a model's parameters.

## Case study: Application to a complex physiological model of acute inflammation

We demonstrate our algorithm for discovering parameters of parameterized probabilistic systems on a physiological model that describes the acute inammatory response (AIR) to the administration of the Gram-negative Bacterial endotoxin lipopolysaccharide (LPS) [17]. Our aim is to discover the schedule and doses of LPS that make the model exhibit desired behavioral properties.

We synthesized *twenty eight model parameters*, for a set of *four *specifications given to us by experts with extensive experience with the model. Simulations were performed using the SPARK (Simple Platform for Agent-based Representation of Knowledge) agent-based modeling and simulation framework [54,53]. We describe both the (natural language) expert specifications and their translations into BLTL:

1 There exists a low dose of the lipopolysaccharide (LPS) that stimulates an episode of inflammation which eventually resolves - the system returns to baseline.

Formal specification: $D_{L}\to F^{\delta_{1}}\left(I\wedge F^{\delta_{2}}\left(N\right)\right)$.

2 There exists a high dose of LPS that stimulates an episode of inflammation which does not resolve, i.e. the system reaches levels of inflammation from which there is no recovery.

Formal specification: $D_{H}\to F^{\delta_{3}}\left(G^{\delta_{4}}I_{H}\right)$.

3 *Desensitization*: For a certain time interval, when one administration of LPS is followed by a second administration of the same dose, the inflammatory response resulting from the second administration is lesser than that from the first.

Formal specification: $D\to F^{\delta_{5}}\left(I_{L}\wedge F^{\delta_{6}}\left(D\to F^{\delta_{7}}I_{H}\right)\right)$.

4 *Priming *: For a certain time interval, when one administration of LPS is followed by a second administration of the same dose, the inflammatory response resulting from the second administration is greater than that from the first.

Formal specification: $D\to F^{\delta_{8}}\left(I_{H}\wedge F^{\delta_{9}}\left(D\to F^{\delta_{10}}I_{L}\right)\right)$.

*D_L _*(*D_H_*) represents a low (high) dose of LPS, *D *is a dose of unknown magnitude, but likely to be neither too low nor very high, *I *indicates that an inammatory event occurred, *N *is the event of entering a non-inammatory state, and *I_L _*(*I_H_*) stands for lower (higher) level of inammation. Also, ∀i∈{1…10}, $\delta_{i}\in \mathbb{N}$ represents the (discrete) simulation time steps between the relevant events. For initial values of each of the parameters, we used a randomly chosen value within bounds provided as part of the specification.

We *successfully synthesized 28 parameters *(shown in Table 1) for the acute inflammatory response model against four behavioral specifications. Our Bayesian model checking-based parameter synthesis algorithm took *less than 24 hours to synthesize this parameter set *on a 1400 MHz, 64-core machine running the Linux operating system.

**Table 1:** Parameters of the acute inflammatory response model synthesized by our algorithm.

param. 1 (LPS-evap)	0.932575
param. 2 (mac-act-LPS)	0.661416
param. 3 (mac-act-pro)	0.326682
param. 4 (mac-regen)	12.655
param. 5 (mac-age)	60.5967
param. 6 (mac-act-dam)	0.3916
param. 7 (max-pro-dam)	18.5986
param. 8 (pro-dam-thresh)	0.51023
param. 9 (damage-evap)	0.276594
param. 10 (anti-heal-thresh)	7.92487
param. 11 (mac-anti)	0.442621
param. 12 (anti-evap)	0.623503
param. 13 (pro-evap)	0.142298
param. 14 (mac-prop)	8.39519
param. 15 (exp1-dose-time)	149.574
param. 16 (exp1-dose-duration)	4.80575
param. 17 (exp1-dose-amount)	3352.54
param. 18 (exp2-dose-time)	467.262
param. 19 (exp2-dose-duration)	458.451
param. 20 (exp2-dose-amount)	896067
param. 21 (exp3-1st-dose-time)	33.3838
param. 22 (exp3-2nd-dose-time)	407.352
param. 23 (exp3-doses-duration)	41.6759
param. 24 (exp3-doses-amount)	2628.97
param. 25 (exp4-1st-dose-time)	8.24293
param. 26 (exp4-2nd-dose-time)	411.959
param. 27 (exp4-doses-duration)	4.40842
param. 28 (exp4-doses-amount)	4494.65

**Figure 2:**
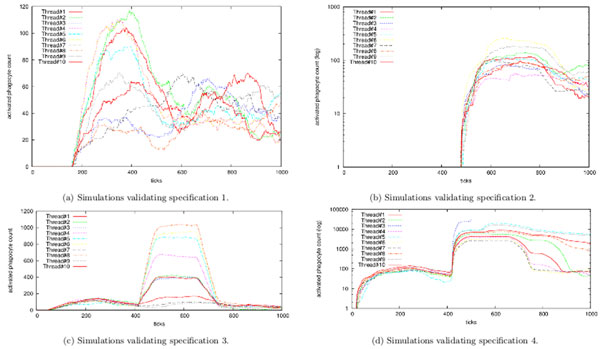
**Parallel simulations showing SPARK output for 10 threads**. Figures (a), (b), (c), and (d) show traces for the activated phagocyte count over time on invocation of the ABM simulator. Satisfaction of the four specifications was determined by a monitoring script that checks traces for each of the desired behaviors. One can visually verify that the ABM parameterized with the values in Table 1 satisfies all the four expert-provided specifications.

Figure 2 shows the satisfaction of all four specifications by depicting model simulations when parameterized at the synthesized parameter set from Table 1. The first 14 parameters are fundamental to the model and denote various attributes that cannot be measured experimentally. The remaining 14 parameters describe the endotoxin administration schedule. Of these, the first 3 parameters indicate the case where inflammatory event occurs but is later resolved; the next 3 parameters show the scenario where an inflammatory event occurs that is never resolved. Both of these scenarios are demonstrated on the same fundamental inflammation model.

The last 4 parameters denote the *priming *scenario (specification 3), and the second-last set of 4 parameters denotes the *desensitization *scenario (specification 4). Both priming and desensitization are phenomena in which repeated administration of endotoxin leads to either an augmented (priming) or reduced (desensitization) level of inflammation as compared to a single administration of endotoxin [17,78-81].

We conclude that a low dose for a high duration causes priming behavior whereas an even lower dose administered for a short period of time results in desensitization. Thus, our algorithm synthesizes a model that demonstrates all four behavioral properties (i.e. specifications 1, 2, 3, 4).

## Discussion

Probabilistic computational models have been to used to analyze complex phenomena in areas that include the study of global ecology, forced migration, the spread of infectious diseases and threats to international security arising from local and regional conflicts [48,82-84].

While the use of mathematical models for computer simulations in systems biology is not new, recent trends show a marked qualitative change in the nature of the models, with the explosive growth in the use of agent-based models because of their natural ability to represent multi-scale biological systems. Agent-based models use a set of interaction rules among individual components of the system that have a spatial location. These models are therefore suited for describing event-based, non-deterministic and highly parallel systems. Like for other stochastic models, discovering parameter values of ABMs in a way that makes the model conform to actual experimental data is an ongoing research challenge.

In recent years, there have been attempts to automate the discovery of model parameters using modern high-performance computing techniques. The ongoing exponential increase in computational power provides an opportunity for building software that can automatically find parameter values of complex stochastic models, given specifications describing the relevant properties the completed model should ideally have.

We described a new algorithmic technique for parameter synthesis for agent-based models that uses Bayesian model checking and simulated annealing to find a model that meets expert-provided behavioral specifications. We applied our algorithm to discover twenty-eight parameters in a model of the acute inflammatory response in humans written in the SPARK modeling framework.

## Future work

We plan to pursue several directions for future work:

• The current sampling strategy of drawing a fresh set of samples for every parameter perturbation may be avoided by employing change of measures arguments and reusing earlier samples [85].

• The construction of expert insight from heterogeneous, uncertain data sets has not been discussed. We plan to automate the process of *learning specifications *from time-series data, which will allow researchers to build better models using specifications generated automatically from experimental data.

• After comparing different parameter estimation techniques for stochastic models in systems biology, we are convinced of the need to develop open benchmarks of computational models and experimental datasets (like time-series data) that would help evaluate existing and proposed solutions to the parameter estimation problem.

• We plan to study the problem of parameter sensitivity, i.e. ensuring that parameter values discovered should be robust enough so that a slight change in them does not cause drastically different model behavior. This problem is especially important in biology because experimental data are often not only sparse but also contains measurement errors [45].

• Finally, the users of search algorithms are always concerned about the issue of scalability, i.e. whether or not the technique would work efficiently when the problem size is large, resulting in a high-dimensional parameter space. To address this issue, we plan to investigate various model reduction techniques [86].

## Competing interests

The authors declare that they have no competing interests.

## Authors' contributions

FH designed the parameter discovery algorithm for probabilistic parameterized computational models. CJL and SKJ designed algorithms for Bayesian statistical model checking. QM led the design and implementation of the SPARK framework for simulation of agent-based models. JDM wrote the acute inflammatory response (AIR) model in the SPARK programming language (SPARK-PL). YV developed the physiological model of the acute inflammation due to trauma and hemorrhagic shock (T/HS). All authors contributed to the design of experiments. SKJ directed the overall project. All authors read and approved the final manuscript.

## Declarations

Publication charges for this article have been funded by Carnegie Mellon University (NIH grant P41 GM103712 to CJL).

This article has been published as part of *BMC Bioinformatics *Volume 16 Supplement 17, 2015: Selected articles from the Fourth IEEE International Conference on Computational Advances in Bio and medical Sciences (ICCABS 2014): Bioinformatics. The full contents of the supplement are available online at http://www.biomedcentral.com/bmcbioinformatics/supplements/16/S17.

## References

[B1] Lin HS Wooley JC Catalyzing Inquiry at the Interface of Computing and Biology 2005 National Academies Press, Washington DC, USA 20669460

[B2] Antoniotti M Policriti A Ugel N Mishra B Model building and model checking for biochemical processes Cell Biochemistry and Biophysics 2003 38 3 271 286 12794268 10.1385/CBB:38:3:271

[B3] Balci O Verification, validation, and testing Handbook of simulation 1998 10 335 393

[B4] Sargent RG Verification and validation of simulation models Journal of Simulation 2013 7 12 24

[B5] Wilkinson DJ Stochastic modelling for quantitative description of heterogeneous biological systems Nature Reviews Genetics 2009 10 2 122 133 10.1038/nrg2509 19139763

[B6] Schwartz R Biological Modeling and Simulation: a Survey of Practical Models, Algorithms, and Numerical Methods 2008 MIT Press, Cambridge, Massachusetts, USA

[B7] Gunawardena J Models in systems biology: the parameter problem and the meanings of robustness Elements of computational systems biology 2010 1

[B8] Baier C Katoen JP Principles of Model Checking vol 26202649 2008 MIT Press, Cambridge, Massachusetts, USA

[B9] Kwiatkowska M Norman G Parker D Stochastic model checking Formal Methods for Performance Evaluation 2007 Springer, Germany 220 270

[B10] An G Mi Q Dutta-Moscato J Vodovotz Y Agent-based models in translational systems biology Wiley Interdisciplinary Reviews: Systems Biology and Medicine 2009 1 2 159 171 20835989 10.1002/wsbm.45 PMC3640333

[B11] Bonabeau E Agent-based modeling: Methods and techniques for simulating human systems Proceedings of the National Academy of Sciences 2002 99 suppl 3 7280 7287 10.1073/pnas.082080899 PMC128598 12011407

[B12] Engl HW Flamm C Kügler P Lu J Müller S Schuster P Inverse problems in systems biology Inverse Problems 2009 25 12 123014

[B13] Banga JR Optimization in computational systems biology BMC systems biology 2008 2 1 47 18507829 10.1186/1752-0509-2-47 PMC2435524

[B14] Emerson EA Temporal and modal logic Handbook of Theoretical Computer Science, Volume B: Formal Models and Sematics (B) 1990 995 1072

[B15] Aarts E Korst J Michiels W Simulated annealing Search Methodologies 2005 Springer, New York 187 210

[B16] Jha SK Clarke EM Langmead CJ Legay A Platzer A Zuliani P A bayesian approach to model checking biological systems Computational Methods in Systems Biology 2009 218 234

[B17] Day J Rubin J Vodovotz Y Chow CC Reynolds A Clermont G A reduced mathematical model of the acute inflammatory response ii. capturing scenarios of repeated endotoxin administration Journal of theoretical biology 2006 242 1 237 256 16616206 10.1016/j.jtbi.2006.02.015

[B18] Sun J Garibaldi JM Hodgman C Parameter estimation using metaheuristics in systems biology: a comprehensive review Computational Biology and Bioinformatics, IEEE/ACM Transactions 2012 9 1 185 202 10.1109/TCBB.2011.63 21464505

[B19] Gonzalez OR Küper C Jung K Naval PC Mendoza E Parameter estimation using simulated annealing for s-system models of biochemical networks Bioinformatics 2007 23 4 480 486 17038344 10.1093/bioinformatics/btl522

[B20] Simulated Annealing http://mathworld.wolfram.com/SimulatedAnnealing.html [From MathWorld-A Wolfram Web Resource, created by Eric W. Weisstein]

[B21] Lillacci G Khammash M Parameter estimation and model selection in computational biology PLoS computational biology 2010 6 3 1000696 10.1371/journal.pcbi.1000696 PMC2832681 20221262

[B22] Reinker S Altman R Timmer J Parameter estimation in stochastic biochemical reactions IEE Proceedings-Systems Biology 2006 153 4 168 178 16986618 10.1049/ip-syb:20050105

[B23] Rodriguez-Fernandez M Mendes P Banga JR A hybrid approach for efficient and robust parameter estimation in biochemical pathways Biosystems 2006 83 2 248 265 16236429 10.1016/j.biosystems.2005.06.016

[B24] Moles CG Mendes P Banga JR Parameter estimation in biochemical pathways: a comparison of global optimization methods Genome research 2003 13 11 2467 2474 14559783 10.1101/gr.1262503 PMC403766

[B25] Koh G Teong HFC Clément MV Hsu D Thiagarajan P A decompositional approach to parameter estimation in pathway modeling: a case study of the akt and mapk pathways and their crosstalk Bioinformatics 2006 22 14 271 280 10.1093/bioinformatics/btl264 16873482

[B26] Torres A Bentley T Bartels J Sarkar J Barclay D Namas R Constantine G Zamora R Puyana JC Vodovotz Y Mathematical modeling of posthemorrhage inflammation in mice: studies using a novel, computer-controlled, closed-loop hemorrhage apparatus Shock 2009 32 2 172 178 19008782 10.1097/SHK.0b013e318193cc2b

[B27] Mathew S Bartels J Banerjee I Vodovotz Y Global sensitivity analysis of a mathematical model of acute inflammation identifies nonlinear dependence of cumulative tissue damage on host interleukin-6 responses Journal of theoretical biology 2014 358 132 148 24909493 10.1016/j.jtbi.2014.05.036 PMC4125477

[B28] Donzé A Clermont G Langmead CJ Parameter synthesis in nonlinear dynamical systems: Application to systems biology Journal of Computational Biology 2010 17 3 325 336 20377448 10.1089/cmb.2009.0172

[B29] Reynolds A Rubin J Clermont G Day J Vodovotz Y Bard Ermentrout G A reduced mathematical model of the acute inflammatory response: I. derivation of model and analysis of anti-inflammation Journal of theoretical biology 2006 242 1 220 236 16584750 10.1016/j.jtbi.2006.02.016

[B30] Kumar R Clermont G Vodovotz Y Chow CC The dynamics of acute inflammation Journal of Theoretical Biology 2004 230 2 145 155 15321710 10.1016/j.jtbi.2004.04.044

[B31] Calzone L Chabrier-Rivier N Fages F Soliman S Machine learning biochemical networks from temporal logic properties Transactions on Computational Systems Biology VI 2006 Springer, Germany 68 94

[B32] Clarke E Fehnker A Jha SK Veith H Temporal logic model checking Handbook of Networked and Embedded Control Systems 2005 Birkhauser, Boston, USA 539 558

[B33] Jarrah AS Laubenbacher R Stigler B Stillman M Reverse-engineering of polynomial dynamical systems Advances in Applied Mathematics 2007 39 4 477 489

[B34] Dreossi T Dang T Parameter synthesis for polynomial biological models Proceedings of the 17th International Conference on Hybrid Systems: Computation and Control 2014 ACM 233 242

[B35] Batt G Page M Cantone I Goessler G Monteiro P De Jong H Efficient parameter search for qualitative models of regulatory networks using symbolic model checking Bioinformatics 2010 26 18 603 610 20823328 10.1093/bioinformatics/btq387 PMC2935427

[B36] Cantone I Marucci L Iorio F Ricci MA Belcastro V Bansal M Santini S Di Bernardo M Di Bernardo D Cosma MP A yeast synthetic network for in vivo assessment of reverse-engineering and modeling approaches Cell 2009 137 1 172 181 19327819 10.1016/j.cell.2009.01.055

[B37] Donaldson R Gilbert D A model checking approach to the parameter estimation of biochemical pathways Computational Methods in Systems Biology 2008 Springer 269 287

[B38] Rizk A Batt G Fages F Soliman S On a continuous degree of satisfaction of temporal logic formulae with applications to systems biology Computational Methods in Systems Biology 2008 Springer 251 268

[B39] Mancini T Tronci E Salvo I Mari F Massini A Melatti I Computing biological model parameters by parallel statistical model checking Bioinformatics and Biomedical Engineering 2015 Springer, Switzerland 542 554

[B40] Shack W Tam P Lardner T A mathematical model of the human menstrual cycle Biophysical journal 1971 11 10 835 5132945 10.1016/S0006-3495(71)86257-4 PMC1484038

[B41] Ditlevsen S Samson A Introduction to stochastic models in biology Stochastic Biomathematical Models 2013 Springer, Germany 3 35

[B42] Allen LJ An Introduction to Stochastic Processes with Applications to Biology 2010 CRC Press, Boca Raton, Florida, USA

[B43] Kwiatkowska M Norman G Parker D Using probabilistic model checking in systems biology ACM SIGMETRICS Performance Evaluation Review 2008 35 4 14 21

[B44] Fisher J Henzinger TA Executable cell biology Nature biotechnology 2007 25 11 1239 1249 10.1038/nbt1356 17989686

[B45] Kitano H Systems biology: a brief overview Science 2002 295 5560 1662 1664 11872829 10.1126/science.1069492

[B46] Mazur J Kaderali L The importance and challenges of Bayesian parameter learning in systems biology Model Based Parameter Estimation 2013 Springer, Germany 145 156

[B47] Laubenbacher R Jarrah AS Mortveit HS Ravi S Agent based modeling, mathematical formalism for Encyclopedia of Complexity and Systems Science 2009 160 176

[B48] Macal CM North MJ Tutorial on agent-based modelling and simulation Journal of Simulation 2010 4 3 151 162

[B49] Calvez B Hutzler G Parameter space exploration of agent-based models Knowledge-Based Intelligent Information and Engineering Systems 2005 Springer 633 639

[B50] Alfarano S Lux T Wagner F Estimation of agent-based models: the case of an asymmetric herding model Computational Economics 2005 26 1 19 49

[B51] Gilli M Winker P A global optimization heuristic for estimating agent based models Computational Statistics & Data Analysis 2003 42 3 299 312

[B52] An G Concepts for developing a collaborative in silico model of the acute inflammatory response using agent-based modeling Journal of critical care 2006 21 1 105 110 16616634 10.1016/j.jcrc.2005.11.012

[B53] Simple Platform for Agent-based Representation of Knowledge (SPARK) http://www.pitt.edu/~cirm/spark/Accessed: 2015-06-23

[B54] Solovyev A Mikheev M Zhou L Dutta-Moscato J Ziraldo C An G Vodovotz Y Mi Q SPARK: A Framework for Multi-Scale Agent-Based Biomedical Modeling International Journal of Agent Technologies and Systems 2010 2 3 18 30 24163721 10.4018/jats.2010070102 PMC3806198

[B55] Solovyev A Mi Q Tzen YT Brienza D Vodovotz Y Hybrid Equation/Agent-Based Model of Ischemia-Induced Hyperemia and Pressure Ulcer Formation Predicts Greater Propensity to Ulcerate in Subjects with Spinal Cord Injury PLoS Comput Biol 2013 9 5 10.1371/journal.pcbi.1003070 PMC3656105 23696726

[B56] Dutta-Moscato J Solovyev A Mi Q Nishikawa T Soto-Gutierrez A Fox IJ Vodovotz Y A multiscale agent-based in silico model of liver fibrosis progression Frontiers in Bioengineering and Biotechnology 2014 2 18 10.3389/fbioe.2014.00018 PMC4126446 25152891

[B57] Ziraldo C Solovyev A Allegretti A Krishnan S Henzel MK Sowa GA Brienza D An G Mi Q Vodovotz Y A computational, tissue-realistic model of pressure ulcer formation in individuals with spinal cord injury Journal of Critical Care 2013 28 1 10.1371/journal.pcbi.1004309 PMC4482429 26111346

[B58] Finkbeiner B Sipma H Checking finite traces using alternating automata Formal Methods in System Design 2004 24 2 101 127

[B59] Thati P Rosu G Monitoring algorithms for metric temporal logic specifications Electr Notes Theor Comput Sci 2005 113 145 162

[B60] Jha SK Model validation and discovery for complex stochastic systems 2010 PhD thesis, Carnegie Mellon University

[B61] Legay A Delahaye B Bensalem S Statistical model checking: An overview Runtime Verification 2010 Springer 122 135

[B62] Zuliani P Platzer A Clarke EM Bayesian statistical model checking with application to stateflow/simulink verification Formal Methods in System Design 2013 43 2 338 367

[B63] Alur R Courcoubetis C Halbwachs N Henzinger TA Ho PH Nicollin X Olivero A Sifakis J Yovine S The algorithmic analysis of hybrid systems Theoretical computer science 1995 138 1 3 34

[B64] Jhala R Majumdar R Software model checking ACM Computing Surveys (CSUR) 2009 41 4 21

[B65] Aziz A Sanwal K Singhal V Brayton R Model-checking continuous-time markov chains ACM Transactions on Computational Logic (TOCL) 2000 1 1 162 170

[B66] Baier C Haverkort B Hermanns H Katoen JP Model-checking algorithms for continuous-time markov chains Software Engineering, IEEE Transactions 2003 29 6 524 541

[B67] Hérault T Lassaigne R Magniette F Peyronnet S Approximate probabilistic model checking Verification, Model Checking, and Abstract Interpretation 2004 Springer 73 84

[B68] Grosu R Smolka SA Monte carlo model checking Tools and Algorithms for the Construction and Analysis of Systems 2005 Springer, Germany 271 286

[B69] Sen K Viswanathan M Agha G Statistical model checking of black-box probabilistic systems Computer Aided Verification 2004 Springer 202 215

[B70] Younes HLS Simmons RG Statistical probabilistic model checking with a focus on time-bounded properties Inf Comput 2006 204 9 1368 1409

[B71] Younes HLS Simmons RG Brinksma, E., Larsen, K.G Probabilistic verification of discrete event systems using acceptance sampling CAV Lecture Notes in Computer Science 2002 2404 Springer, Germany 223 235

[B72] Wald A Sequential Analysis 1973 Dover, Courier Corporation, USA

[B73] Langmead CJ Generalized Queries and Bayesian Statistical Model Checking in Dynamic Bayesian Networks: Application to Personalized Medicine Proc of the 8th International Conference on Computational Systems Bioinformatics (CSB) 2009 201 212

[B74] Bertsimas D Tsitsiklis J Simulated annealing Statistical Science 1993 10 15

[B75] Wald A Sequential tests of statistical hypotheses The Annals of Mathematical Statistics 1945 16 2 117 186

[B76] Faraz Hussain Raj Gautam Dutta Sumit Kumar Jha Christopher James Langmead Susmit Jha Istrail, S., Mandoiu, I.I., Pop, M., Rajasekaran, S., Spouge, J.L Parameter discovery for stochastic biological models against temporal behavioral specifications using an SPRT based Metric for simulated annealing ICCABS 2012 IEEE Computer Society, USA 1 6

[B77] Hussain Faraz Jha Sumit K Jha Susmit Langmead Christopher J Parameter discovery in stochastic biological models using simulated annealing and statistical model checking International Journal of Bioinformatics Research and Applications 2014 10 4/5 519 539 24989866 10.1504/IJBRA.2014.062998 PMC4438994

[B78] Rivière B Epshteyn Y Swigon D Vodovotz Y A simple mathematical model of signaling resulting from the binding of lipopolysaccharide with toll-like receptor 4 demonstrates inherent preconditioning behavior Mathematical biosciences 2009 217 1 19 26 18950647 10.1016/j.mbs.2008.10.002 PMC2651675

[B79] Foteinou P Calvano S Lowry S Androulakis I Modeling endotoxin-induced systemic inflammation using an indirect response approach Mathematical biosciences 2009 217 1 27 42 18840451 10.1016/j.mbs.2008.09.003 PMC3045970

[B80] An G A model of tlr4 signaling and tolerance using a qualitative, particle-event-based method: Introduction of spatially configured stochastic reaction chambers (scsrc) Mathematical biosciences 2009 217 1 43 52 18950646 10.1016/j.mbs.2008.10.001

[B81] Dong X Foteinou PT Calvano SE Lowry SF Androulakis IP Agent-based modeling of endotoxin-induced acute inflammatory response in human blood leukocytes PloS one 2010 5 2 9249 10.1371/journal.pone.0009249 PMC2823776 20174629

[B82] Farmer JD Foley D The economy needs agent-based modelling Nature 2009 460 7256 685 686 19661896 10.1038/460685a

[B83] Edwards S Computational tools in predicting and assessing forced migration Journal of Refugee Studies 2008 21 3 347 359

[B84] El-Sayed AM Scarborough P Seemann L Galea S Social network analysis and agent-based modeling in social epidemiology Epidemiologic Perspectives & Innovations 2012 9 1 1 22296660 10.1186/1742-5573-9-1 PMC3395878

[B85] Jha SK Langmead CJ Exploring behaviors of sde models of biological systems using change of measures Computational Advances in Bio and Medical Sciences (ICCABS), 2011 IEEE 1st International Conference 2011 IEEE 111 116

[B86] Benner P Gugercin S Willcox K A survey of model reduction methods for parametric systems Preprint MPIMD/13-14, Max Planck Institute Magdeburg (August 2013). Available from http://www.mpi-magdeburg.mpg.de/preprints/

